# Supplementary material for: The Proteomic Landscape of Resting and Activated CD4+ T Cells Reveal Insights into Cell Differentiation and Function
Source: Int J Mol Sci. 2020 Dec 29;22(1):275. doi: 10.3390/ijms22010275 (PMC7795831; doi:10.3390/ijms22010275)
Supplement: Supplementary file 1 [file ijms-22-00275-s001.zip › Supplementary_data_Legends.pdf]

Supplementary material

Supplementary figures

Supplementary Figure 1. A & B. Volcano plots depicting upregulated and downregulated proteins in Donor 1 and Donor 2 CD4+ T cells in response to activation.

Supplementary Figure 2 Enriched Biological Processes from proteins downregulated in **A.** Donor 1 and **B.** Donor 2 in response to activation

Supplementary Figure 3. Protein expression profiles of various protein classes in resting and activated CD4+ T cells. Heatmaps depicting intensity-based abundances for differential expressed proteins for A. Adaptive immune response B. Hypoxia markers C. Interleukins D. Other cytokines E. MAP kinases F. Markers of Reactive Oxygen Species (ROS)

Supplementary Figure 4. Protein expression profiles for metabolic pathways in resting and activated CD4+ T cells. Heatmaps depicting intensity-based abundances for differential expressed proteins for A. Glycolysis/Gluconeogenesis B. Amino acid metabolism C. Lipid metabolism D. Oxidative Phosphorylation

Supplementary Figure 5 Protein expression profiles for cellular processes in resting and activated CD4+ T cells. Heatmaps depicting intensity-based abundances for differential expressed proteins for A. Cell cycle markers B. Apoptosis C. Autophagy D. Phagocytosis

Supplementary tables

Supplementary Table 1. Proteins identified from Activated and Resting CD4+ T cells using MaxQuant and significantly changing proteins in Activated and Resting CD4+ T cells in Donor 1 and 2

Supplementary Table 2. Node properties of Cytoscape network generated from protein upregulated in activated primary CD4+ T cells

Supplementary Table 3. Proteins identified from proteomic analysis of SUP-T1 cells

Supplementary Table 4. Lists of proteins identified in Resting primary CD4+ T cells, SUP-T1 and Jurkat cells (Wu *et al.* (2007))

Supplementary Table 5. Comparison of current human CD4+ T-cell proteomics dataset with mouse CD4+ T cell proteomics data (Howden *et al.*)

Supplementary Table 6. Comparison of current CD4+ T-cell proteomics dataset with previous proteomic studies

Supplementary Table 7. Lists of proteins identified from the current study and previous studies on CD4+ T-cell proteomics
